# Oxidative Stress Mechanisms Caused by Ag Nanoparticles (NM300K) are Different from Those of AgNO_3_: Effects in the Soil Invertebrate *Enchytraeus crypticus*

**DOI:** 10.3390/ijerph120809589

**Published:** 2015-08-14

**Authors:** Maria J. Ribeiro, Vera L. Maria, Janeck J. Scott-Fordsmand, Mónica J. B. Amorim

**Affiliations:** 1 Department of Biology & CESAM, University of Aveiro, Aveiro 3810-193, Portugal; E-Mails: mariajribeiro@ua.pt (M.J.R.); vmaria@ua.pt (V.L.M.); 2 Department of Bioscience, Aarhus University, Vejlsovej 25, Silkeborg DK-8600, Denmark; E-Mail: jsf@bios.au.dk

**Keywords:** antioxidant system, reactive oxygen species, metallothionein, lipid damage, soil

## Abstract

The mechanisms of toxicity of Ag nanoparticles (NPs) are unclear, in particular in the terrestrial environment. In this study the effects of AgNP (AgNM300K) were assessed in terms of oxidative stress in the soil worm *Enchytraeus crypticus*, using a range of biochemical markers [catalase (CAT), glutathione peroxidase (GPx), glutathione S-transferase (GST), glutathione reductase (GR), total glutathione (TG), metallothionein (MT), lipid peroxidation (LPO)]. *E. crypticus* were exposed during 3 and 7 days (d) to the reproduction EC_20_, EC_50_ and EC_80_ levels of both AgNP and AgNO_3_. AgNO_3_ induced oxidative stress earlier (3 d) than AgNP (7 d), both leading to LPO despite the activation of the anti-redox system. MT increased only for AgNP. The Correspondence Analysis showed a clear separation between AgNO_3_ and AgNP, with e.g., CAT being the main descriptor for AgNP for 7 d. LPO, GST and GPx were for both 3 and 7 d associated with AgNO_3_, whereas MT and TG were associated with AgNP. These results may reflect a delay in the effects of AgNP compared to AgNO_3_ due to the slower release of Ag^+^ ions from the AgNP, although this does not fully explain the observed differences, *i.e*., we can conclude that there is a nanoparticle effect.

## 1. Introduction

In addition to their bactericidal properties, silver nanoparticles (AgNPs) have been reported to cause *in vitro* and *in vivo* effects in organisms other than bacteria, e.g. in vertebrates [*Danio rerio* and *Mus musculus* [1,2] and invertebrates [*Drosophila melanogaster*, *Caenorhabditis elegans* and *Eisenia fetida* [3,4,5]. AgNP toxicity has been mostly attributed to the generation of reactive oxygen species (ROS) in cells [3,6,7]. Park *et al*. [8] proposed that AgNPs’ toxicity may partly follow Trojan-horse type mechanisms *i.e*., AgNP are taken up by the cell as particles and dissolution occurs inside the cell causing a high local concentration of Ag^+^, however there may also be an external dissolution of AgNP with subsequent ion- related Ag^+^ toxicity. Hence, it seems that both particulate and ion-based mechanism of action may be at play, as further discussed by other authors [9,10,11].

Few studies have been performed on terrestrial oligochaetes. For example, time-response tests with *Eisenia andrei* [5], a 7-day test with *E. fetida* [12], or the growth and survival test with *Lumbricus rubellus* [13]. Recently, a study on the oxidative stress response in *E. fetida* [14] comparing short- and longer-term (4 and 28 days) exposure period showed a time dependent response and differences in the redox mechanisms sequence between salt- and nano-Ag salt. Despite the activation of the anti-redox enzymes for both Ag forms, lipid peroxidation (LPO) occurred for longer-term exposures. Further, Hayashi *et al*. [5] showed a faster induction of oxidative stress markers in *E. fetida* when exposed to Ag salt than when exposed to AgNPs. More comprehensive differential gene expression response was studied in *Enchytraeus albidus* [11], linking short to longer term effect, *i.e*., gene and reproduction. The authors highlight that testing of AgNPs seems to require longer exposure period to be comparable in terms of effect/risk assessment with other chemicals. 

In the present study we aimed to characterize the antioxidant system in the omnipresent standard soil species *Enchytraeus crypticus* [15,16,17] exposed to AgNP. This involved the exposure to dispersed AgNPs (NM300K, JRC standard particles) and Ag salt (AgNO_3_) at the reproduction effect concentrations (EC_20_, EC_50_ and EC_80_) during a short time series exposure: 0, 3 and 7 days. A set of oxidative stress biomarkers was used, which included catalase (CAT), glutathione peroxidase (GPx), glutathione reductase (GR) and glutathione-S-transferase (GST) activities, the total glutathione (TG), metallothionein (MT) and LPO levels.

## 2. Experimental Section

### 2.1. Test Organism

*Enchytraeus crypticus* (Oligochaeta: Enchytraeidae) were maintained under laboratory conditions, 19 ± 1 °C and 16: 8 h (light: dark) photoperiod regime in agar plates, consisting of a sterile mixture of four solutions (CaCl_2_·2H_2_O, MgSO_4_, KCl, NaHCO_3_) and Bacti-Agar medium as a substrate. The animals were fed on autoclaved dried oats. Adult organisms with visible clitellum and similar size were selected for the experiment.

### 2.2. Test Materials 

The AgNO_3_ (high-grade, 98.5–99.9% purity) was purchased from Sigma–Aldrich (St. Louis, MO, USA). The silver nanoparticles (AgNPs) used were the fully characterized standard representative material AgNM300K from the European Commission Joint Research Centre (JRC) [18]. The AgNM300K is dispersed in 4% polyoxyethylene glycerol triolaete and polyoxyethylene (20) sorbitan mono-laurate (Tween 20), thus the dispersant was also tested alone.

### 2.3. Test Soil and Spiking

Natural standard soil LUFA 2.2 (Speyer, Rhineland-Palatinate, Germany) was used and has the following main characteristics: grain size distribution of 7.2% clay, 8% silt and 77.5% sand, pH (CaCl_2_) = 5.5, water holding capacity (WHC) of 45 g/100 g, a cation exchange capacity (CEC) of 10 meq/100 g, and an organic carbon (OC) content of 1.77%.

The soil was dried (72 h, 80 °C) before use. Spiking was performed as aqueous solution onto pre-moistened soil and homogeneously mixed. The soil was left to equilibrate for 3 days prior test start. For AgNP spiking was performed individually per replicate, for AgNO_3_ was done per concentration batch. Test concentrations used (Table 1) corresponded to the EC_20_, EC_50_ and EC_80_ for reproductive effects. The control soil was prepared by adding deionized water to adjust to the adequate moisture content (50% of the WHC max). A control dispersant was also performed by adding the equivalent to the maximum dispersant volume as added with the AgNP spiking.

**Table 1:** Test exposure concentrations of AgNO_3_ and AgNP, corresponding to the estimated reproduction effect concentrations (ECs), expressed as mg Ag / kg soil dry weight.

Test material	EC_20_	EC_50_	EC_80_
AgNO_3_	45	60	96
AgNP (NM 300K)	60	170	225

### 2.4. Test Procedures 

Test procedures followed the OECD standard Enchytraeid Reproduction Test (ERT) guideline 220 [16] with adaptations. In short, each replicate consisted of a glass vessel (Ø 4 cm, 45 mL volume) containing 20 g soil ww (wet weight) with food supply. Fifty organisms were added in each test vessel and covered with a lid with small holes. Test conditions were 20 ± 1 °C and 16: 8 h photoperiod. Five replicates per treatment were used. At each sampling time (0, 3 and 7 days), organisms were carefully collected from soil, rinsed in water, introduced into a microtube, weighed, frozen in liquid nitrogen and stored at −80 °C until further analysis. 

### 2.5. Biochemical Analysis

Procedures followed the described for *E. albidus* [19] with adaptations. Pools of 40 organisms were homogenized using an ultrasonic homogenizer (Sonifier 250, Branson Sonicator, Swedesboro, NJ) in 2000 µL of potassium phosphate buffer (0.1 mM, pH 7.4 containing EDTA 1 mM and DTT 1 mM). Part of the homogenate (150 µL) was separately stored (−80 °C) with 2.5 µL BHT (2,6-dibutyloxy-4-methylphenol) 4% in methanol to block tissue oxidation for later lipid peroxidation (LPO) measurement. The rest of the homogenate was centrifuged at 10,000 × g for 20 min at 4 °C and the post mitochondrial supernatant (PMS) was kept at −80 °C for further analysis. The procedures for biochemical analysis (biomarkers) were based on spectrometric methods and a Multiskan Spectrum microplate reader (Thermo Scientific, Swedesboro, NJ) was used. Protein concentration was assayed using the Bradford method [20], adapted from BioRad’s Bradford microassay set up in a 96-well flat bottom plate, using bovine γ-globuline as a standard. Catalase (CAT) activity was measured following the method of Claiborne [21] as described by Giri *et al.* [22]. Changes in absorbance were recorded at 240 nm and CAT activity was calculated in terms of µmol H_2_O_2_ consumed min^−1^ mg^−1^ protein. Glutathione reductase (GR) activity was measured in accordance to the method of Carlberg and Mannervik [23] being quantified by the NADPH loss at 340 nm and expressed as nmol of NADP^+^ formed min^−1^ mg^−1^ protein. Glutathione S-transferase (GST) activity was assessed using 1-chloro-2,4-dinitrobenzene (CDNB) as substrate according to the method of Habig *et al*. [24]. The enzyme activity was recorded at 340 nm and calculated as nmol GS-DNB conjugate min^−1^ mg^−1^ protein.

Total glutathione (TG) levels were measured at 412 nm, using the recycling reaction of reduced glutathione (GSH) with dithionitrobenzoate (DTNB) in the presence of GR excess [25,26]. The results were expressed as nmol of 5-thio-2-nitrobenzoic acid (TNB) formed min^−1^ mg^−1^ protein. LPO occurrence was evaluated according to the procedure of Ohkawa [27] and Bird *et al*. [28], as adapted by Wilhelm Filho *et al*. [29]. Absorbance was measured at 535 nm and results were expressed as µmol of thiobarbituric acid reactive substances (TBARS) formed per milligram of fresh weight. Metallothionein (MT) levels were determined by using the method described by Viarengo *et al*. [30]. Briefly, PMS (500 µL) was added to 95% ethanol with 8% chloroform (500 µL). After mixing it was centrifuged at 6000 × g for 10 min at 4 °C. 50 µL RNA, HCl (10 µL) and cold ethanol (1.2 mL) were added to 700 µL of the supernatant (S6) and frozen for 15 min at −80 °C. After centrifuging at 6000 × g for 1 min (at 4 °C) the supernatant was removed and the pellet was re-suspended in 87% ethanol in 1% chloroform (300 µL). The last centrifuge step was repeated and the pellet was re-suspended in NaCl (150 µL), HCl containing 4 mM ethylenediaminetetraacetic acid (EDTA) (150 µL) and Ellmans reagent (300 µL) with 0.4 mM DTNB, 2 M NaCl and 0.2 M potassium phosphate at pH 8. After 5 min, the absorbance was read at 412 nm. 1 mM GSH in 0.1 M HCl was used as a standard and the amount of MT was expressed as nmol mg^−1^ protein.

### 2.6. Data Analysis

Data was tested for normality (Kolmogorov–Smirnov test) and homogeneity of variance (Levene’s test). Univariate one-way analysis of variance (ANOVA) followed by Dunnetts’ *Post-Hoc* test (*p* < 0.05) [31] was used to test differences between treatments (between day 0 and 3 and 7 and, between control and concentrations).

Multivariate analysis was done to explore the patterns in correlations between the data, using Correspondence Analysis (CA) including all treatments and also when divided per days. The analysis was performed using the software SAS enterprise guide 5.1 [32]. To compensate for the different scales of the biomarkers, the response was normalised using several different normalisation methods all giving the same pattern; in the present normalisation based on averaging is displayed.

## 3. Results

### 3.1. Materials Characterisation

The Ag NM300K silver nanoparticles (AgNPs) from the European Commission Joint Research Centre (JRC) are fully characterized [18]. In short, Ag NM300K are spherical and consist of a colloidal dispersion with a nominal silver content of 10.2 w/w %, dispersed in 4% w/w of polyoxyethylene glycerol trioleate and polyoxyethylene (20) sorbitan mono-laurat (Tween 20), having > 99% number of particles with a nominal size of about 15 nm, with no coating. Transmission Electron Microscopy (TEM) indicated a size of 17 ± 8 nm. Smaller nanoparticles of about 5 nm are also present.

### 3.2. Biological Characterisation

**Figure 1:**
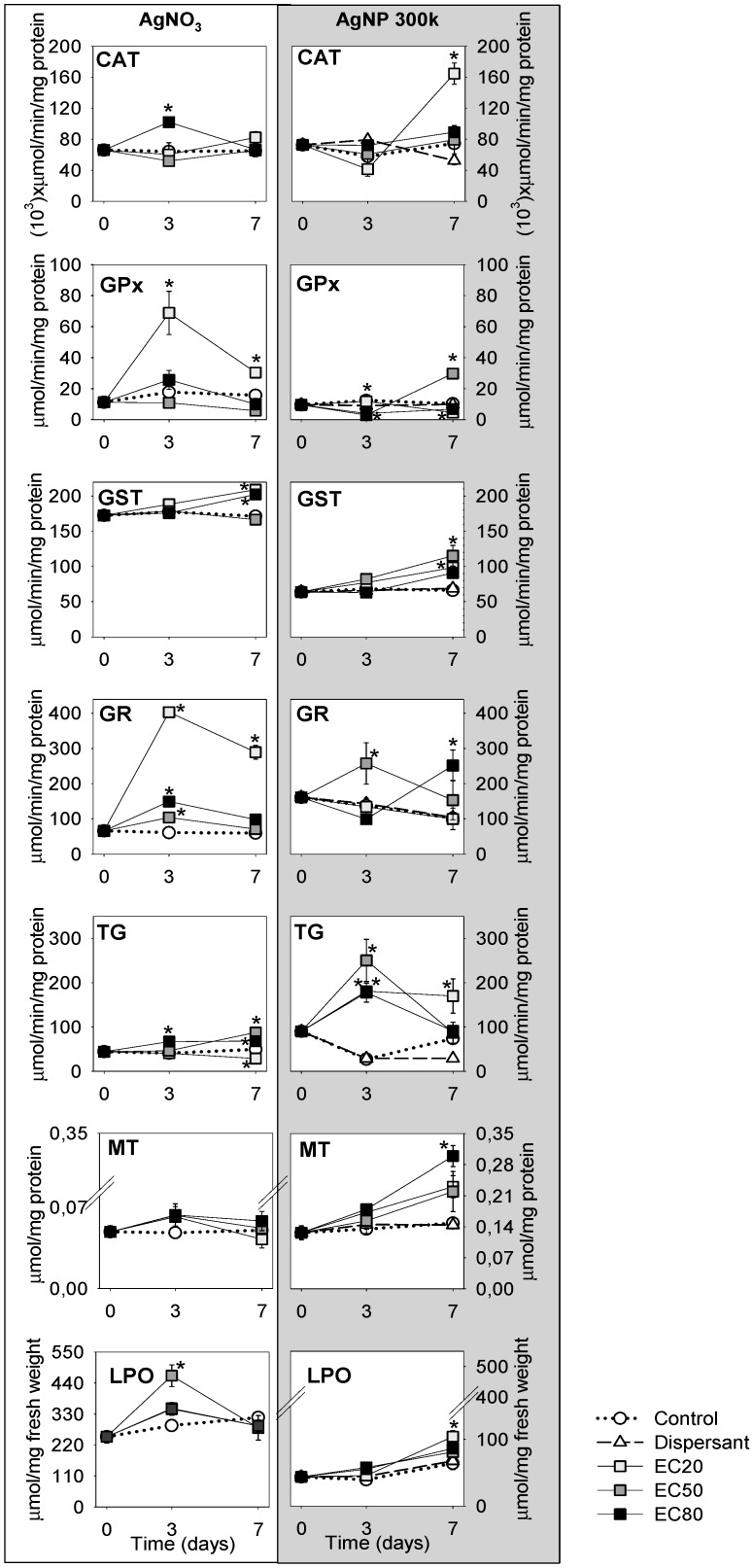
Biochemical analyses results from *Enchytraeus crypticus* exposed to Ag NM300K [60 (EC20), 170 (EC50) and 225 (EC80) mg Ag/kg soil] and AgNO_3_ [45 (EC20), 60 (EC50) and 96 (EC80) mg Ag/kg soil], as sampled at 0, 3–7 days, in terms of catalase (CAT), glutathione peroxidase (GPx), glutathione S-transferase (GST), glutathione reductase (GR), total glutathione (TG), metallothionein (MT) and lipid peroxidation (LPO). Values are expressed as mean ± standard error (n = 5). * (*p* < 0.05, Dunnetts’ test) for differences between control and treatments.

The results obtained for the various biomarkers (univariate) can be depicted in Figure 1.

Summarising (Figure 1), CAT shows a significant increase at the AgNP_EC_20__7d, whereas for AgNO_3_ mostly the EC_80__3d is increased but all is balanced at 7 d. GPx and GR seems to have a bell- shape pattern for the Ag salt between 0–3–7 days, being most pronounced for AgNO_3__EC_20_. For AgNP an increase is observed for GPx at AgNP_EC_50__7d; GR shows a bell shape (0–3–7 d) for EC_50_ exposure and the opposite shape for the EC_80_ one. GST shows a steady increase from 0–7 d for both Ag forms, but with higher absolute values for AgNO_3_ TG shows a bell-shape (0–3–7 d) for AgNP for all ECs whereas for AgNO_3_ changes are minor. MT exhibits a small increase and stabilization for AgNO_3_ along the test period for all tested ECs. For AgNP there is a continuous increase in MT levels from 0–7 days, this being highest for the EC_80__7 d LPO levels are increased for AgNP_EC_20__7 d. For AgNO_3_, LPO levels get back to normal after an increase at day 3 (significantly for EC_50_).

**Figure 2:**
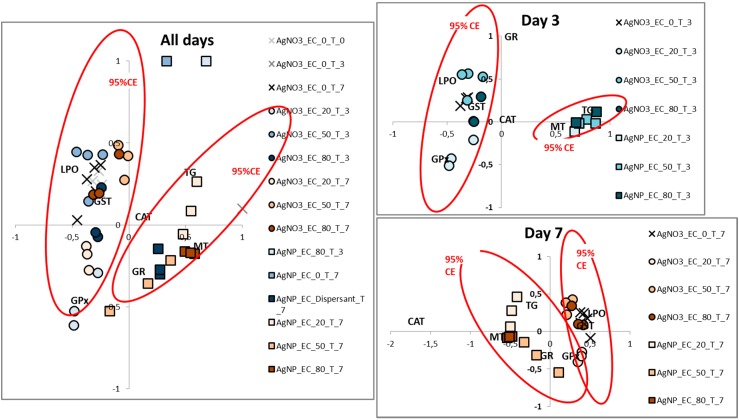
Correspondence analysis of data from *Enchytraeus crypticus* exposed to AgNP (Ag NM300K) [60 (EC_20_), 170 (EC_50_) and 225 (EC_80_) mg Ag/kg soil] and AgNO_3_ [45 (EC_20_), 60 (EC_50_) and 96 (EC_80_) mg Ag/kg soil], as sampled at 0–3–7 days (designated T_0, T_3 and T_7), in terms of catalase (CAT), glutathione peroxidase (GPx), glutathione S-transferase (GST), glutathione reductase (GR), total glutathione (TG), metallothionein (MT) and lipid peroxidation (LPO). CE: confidence ellipses.

The multivariate analysis of the data (Correspondence Analysis) enabled an identification of the overall differences between the AgNO_3_ and AgNP exposures (Figure 2), with a clear separation of the AgNO_3_ and AgNP. It should be noted that whereas Figure 1 shows mean values and standard errors, the multivariate plot displays the individual replicates. When treating the time (d) separately (Figure 2B,C), an even clearer difference was observed. Whereas CAT has no importance for the separation between the exposures on day 3 it was the main descriptor for AgNP for the day 7. LPO, GST and GPx were for both days associated with AgNO_3_, whereas MT and TG were associated with the AgNP over both days.

## 4. Discussion

The results show different biomarker response-patterns for organisms exposed to AgNP and to AgNO_3_, both across all time-points and within each time-point. In the following we first discuss responses to each of the materials and then compare the responses across materials.

### 4.1. AgNO_3_ Mechanisms

At low concentration, GPx was increased during the entire exposure period with a peak response after 3 days. The CAT activity also peaked after 3 days but was only generated by the EC_80_ exposures. Since both CAT and GPx are related to H_2_O_2_ cleavage, it seems that GPx is the initial response, concentration wise, whereas CAT is activated at higher oxidation level (*i.e*., assuming that higher oxidation level is linked to higher exposure concentrations levels). The cooperation between CAT and GPx has been suggested by Baud *et al*. [33] when the action of the enzymes by themselves is not enough for H_2_O_2_ clearance, being both required in the process. Moreover, for higher cellular H_2_O_2_ concentrations, GPx activity is mandatory to avoid CAT inactivation, as also seen in the present experiment. The cellular presence of lipid peroxides and other hydroperoxides can also act as stimulant substrates of GPx. In addition, the lower trend in the GPx activity for higher concentrations (EC_50_ and EC_80_) could reflect the occurrence of hormesis, *i.e*., an overcompensation response to low dose is elicited [34]. This effect has been reported in several studies regarding Ag toxicity, and for both forms [6,35,36,37]. 

The increase in GST activity for EC20 and EC80 may be due to the conjugation of GSH with Ag^+^ ions. Induction in GST genes for both AgNP and Ag^+^ was reported in *E. fetida* after 7 d exposure to 100 mg Ag/kg [38]. A concentration-dependent increase in GST activity was found in *E. fetida* after 14 d exposure to AgNO_3_ [39]. Considering that antioxidant enzyme’s activities didn’t change for EC_50_ at 7 d, but TG values were increased, possibly Ag^+^ were forming complexes with GSH molecules. 

All tested concentrations promptly elevated GR activity, which should increase the GSH levels due to the recycling reaction of GSSG. However, TG content does not reflect this, as an increase in TG levels was only observed for the EC_80_. It is known that GSH can interact with certain metal ions, having high affinity to Ag, [40], hence, it could be directly binding to Ag^+^ ions as a response against oxidative stress, hence although GSH was formed in all concentrations corresponding to an increase in the TG, the formed GSH was biding to Ag+ in the EC_20_ and EC_50_, and the TG being reduced. An increase in GR activity coupled with the occurrence of Ag^+^ chelation by GSH was also proposed for *E. fetida* (75–100 mg Ag/kg, 4 d exposure) [14]. For EC_20_, since GPx activity increased, this enzyme is using GSH to detoxify the cells, also explaining the higher increase in GR activity observed.

Considering the increase in GST and GPx activity for EC_20_ associated with TG content decrease, this must reflect a severe fall in GSH levels, since it’s been mobilized by both enzymes. As to EC_80_, the increase in GST activity and TG levels seems to be a consequence of the conversion of GSH into GSSG. Regarding the higher TG levels (plus the increase in GST and GR activities), we propose two hypotheses/mechanisms for Ag^+^ scavenger: 1) The endogenous GSH was mobilized to scavenge Ag^+^ and/or 2. The increase tendency in MT levels is sufficient to scavenge Ag+ after 3 d. A similar pattern was reported for *Folsomia candida* (EC_50_ of Cu and Cd, 0–10 d exposure), where an induction in MT levels was measured after 6 d followed by a decrease [41]. It is known that Ag has a strong affinity to MT and, since the used method detects unbound MT, MT could had been bound to Ag^+^ before day 3, hence earlier than for AgNP and not detected in the present design. In summary, despite the activated antioxidant mechanisms by *E. crypticus* to avoid oxidative damage, this was not enough to prevent LPO for AgNO_3__EC_50__3d. On the other hand, the mechanism to protect the cells from LPO was more efficient for EC_20_ and EC_80_ during the test period.

### 4.2. AgNPs Mechanisms

For the AgNP exposed organisms, the early decline in CAT and GPx activities could have led to an accumulation of H_2_O_2_ in the cells and contributed to an antioxidant system imbalance seen as enzyme “deactivation/lost” through denaturation process by the ROS build-up inside the cell, *i.e*., AgNP-induced oxidative damage. Nevertheless, CAT and GPx activities increased after 7 d, although for different exposure concentration, which indicates the induction of protein biosynthesis and further activation of other antioxidant enzymes, e.g. GST and GR as observed here, but also an increase in cellular GSH contents which also counteract the ROS action and in this way prevent denaturation of proteins (e.g., enzymes). An increase in GST activity was also observed in *E. fetida* (20–500 mg AgNP/kg, 14 d exposure) [39] and in *Chiromonus riparius* at the mRNA level (0.2–1 AgNP mg/L, 24 hour exposure) [42]. The increase pattern in GST activity is similar between AgNP and Ag salt and could be due to Ag^+^ ions, *i.e*., that AgNP is either dissolved in the media or inside the worm. However there is no clear knowledge of the possible degree of dissolution for Ag NM300K in soils, e.g. van der Ploeg *et al*. [13] report lower Ag in soil pore-water following AgNP exposure compared to AgNO_3_ (without specifying whether this is ions or nanomaterial). In the same study, the AgNP were attached to the worms, with bioaccumulation factors being lower for AgNP exposed worms compared to Ag salt exposed worms. Schlich *et al*. [43] used Diffusion Gradient Thin-films (DGT) and detected equivalent amount of Ag in pore water (it is presently unclear how much AgNP will diffuse into the DGT, as particulate matter is known to enter DGTs) [44]. Considering that a possible detoxification route of Ag^+^ ions involves elimination via conjugation to GSH in a reaction catalysed by GST, this could explain the increase in its activity. Also, it could be acting in response to CAT and GPx failure, which suggest a later action of AgNPs.

While GR activity was induced early, the GST was stable (0–3 d) followed by a later activation for EC_50_ exposure, leading to higher GSH levels mobilized as non-enzymatic antioxidant substrate, functioning by quenching intracellular ROS, albeit it was not sufficient to neutralize them since inhibition in CAT and GPx activities were observed. Consequently a robust increase in the ratio GSH/GSSG content, (as measured by the increase in TG levels) can be possibly due to the presence of GSH. Higher GSH levels were also reported in *E. fetida* [39]. The decrease in TG for EC_50__7d may be related to the GPx and GST increased activities that generated GSSG through GSH oxidation in the cells.

The potential release of Ag^+^ does not seem to be linked to the increase in MT for AgNP_7d given the observations of much lower values for AgNO_3_. An increase in MT mRNA expression was also observed in exposure to AgNP in zebrafish [7] and in *E. fetida* [5,38]. The increase of MT must be linked to the observed TG decrease despite the increase in GST and GR activities. Hence, we consider that the decrease in the ratio GSH/GSSG (implying less available GSH) triggered an increase in the levels of MT (another thiol substrate) in order to reduce the oxidant Ag^+^ action. However, despite these mechanisms, LPO still occurred after 7 d. 

### 4.3. Comparison of Ag Nano and Ag Salt Mechanisms

As seen above, results show different biomarker response-patterns for organisms exposed to AgNP and AgNO_3_, both across all time-points and within each time-point. For AgNO_3_ oxidative stress is associated by an initial activation of enzymes like CAT, GR and GPx, and causing LPO, followed by a decreased importance of GR and GPx at day 7. For AgNP oxidative stress is associated with the increased activities/levels of e.g., GPx, GST, TG but especially CAT from 0–7 d, although the activated mechanisms are not sufficient to avoid LPO as measured at EC_20__7d. The lower LPO at EC_50_ and EC_80_ was possibly related with earlier induction of antioxidant enzymes for these concentrations, however this didn’t occur in AgNO_3_ exposed animals, resulting in higher LPO levels.

It is known that oxidative stress can be directly induced by the active surface of AgNPs [45]. An alternative, may be a combined possibility, is that the present study reflects a delay in the effects of AgNP compared to AgNO_3_ due to the release of Ag^+^ ions from the particles either externally (e.g., in soil) or internally (e.g., in lysosomes), although this is presently impossible to verify for NM300K; it would probably require a soil Bio Ligand Model (BLM) kind of approach to the possible ratio between dissolution and uptake rates. The degree of toxicity caused be the soluble fraction (Ag^+^) and the particulate fraction is unclear and likely vary from experiment to experiment depending on conditions, see e.g., Baalousha *et al*. [46] who argued that not all toxicity is caused by the soluble part. In a research covering the immunological effects of AgNP in human monocytes it was proposed that the release of Ag^+^ generates the production of hydroxyl radicals in acidic endo/lysosomes [47]. Moreover, AgNPs can exert their toxicity by entering the cells and releasing large amounts of Ag^+^, a Trojan-horse type mechanism [8,10]. Hence, the observed effect can partly be due to slower Ag^+^ release from AgNP (outside or inside the organism), which also leads to a change in the order of cascade of events and hence potentiates different effects.

**Figure 3:**
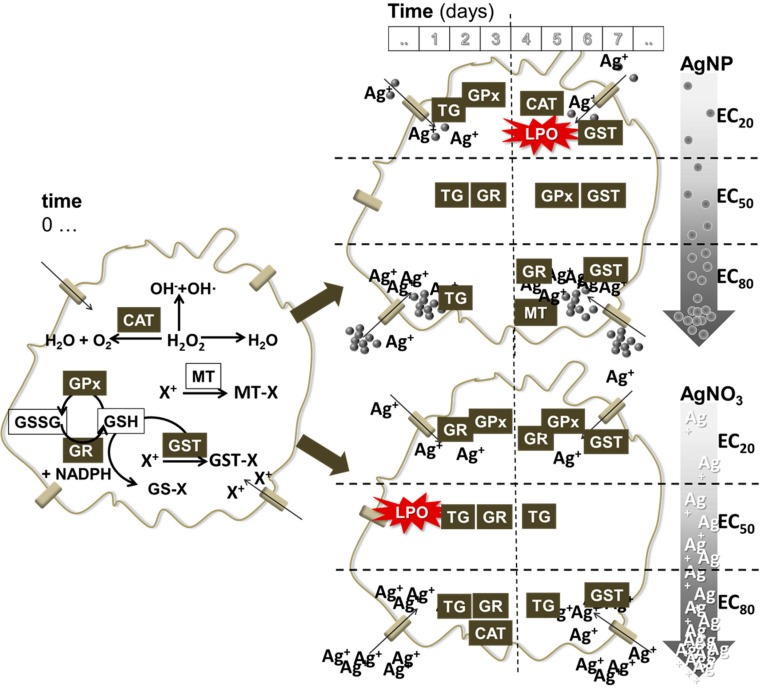
Schematic representation of the redox events in the cell when exposed to AgNP and AgNO_3_ (variation in concentration is signalled in the arrows from low to high (EC20–50–80), top to bottom respectively) and along various time periods (0–3–7 days). Cell at time 0 (left) indicates the general set of existing reactions that occur involving the measured markers in the present study. CAT: catalase, GPx: glutathione peroxidase, GST: glutathione S-transferase, GR: glutathione reductase, TG: total glutathione, MT: metallothionein, LPO: lipid peroxidation.

Figure 3 shows a schematic representation of the events. As can be seen, there are some common features between AgNP and AgNO_3_ in terms of activated enzymes. Variation is observed in terms of time of activation, which by itself can create a different cascade of events. Moreover, the variation can be observed in terms of the induced levels per concentration and per material. Particular differences include e.g., for AgNP the increased CAT and MT.

## 5. Conclusions

Comparison between exposure to AgNP and AgNO_3_ in *E. crypticus* showed dissimilar oxidative stress responses, e.g., a delayed increase in the antioxidant enzymes responses (CAT, GST, GPx) for AgNPs exposure (7 d) compared to AgNO_3_ (3 d), initial LPO damage for AgNO_3_ followed by stabilization, whereas for AgNP LPO occurred after 7 d, MT increased only in organisms exposed to AgNPs. The present can partly reflect a delay in effects of AgNP compared to AgNO_3_ due to the slower release of Ag^+^ ions from the Ag particles, *i.e*., AgNP acts as a continuous source of Ag^+^ to the soil pore-water––although this possible dissolution is still unclear.
